# Mapping the quality of information on osteoporosis: a cross-sectional analysis of online health information

**DOI:** 10.1186/s12891-026-09711-2

**Published:** 2026-03-07

**Authors:** Sandro Zacher, Julia Lauberger, Julia Lühnen, Anke Steckelberg

**Affiliations:** 1 Institute of Health, Midwifery and Nursing Science, Medical Faculty of Martin Luther University Halle-Wittenberg, University Medicine Halle, Halle (Saale), Germany; 2 Institute of Clinical Nursing Science, Charité – Universitätsmedizin Berlin, corporate member of Freie Universität Berlin, Humboldt Universität zu Berlin, Berlin, Germany

**Keywords:** Osteoporosis, Evidence-based health information, Online health information, Consumer health information, Informed decision-making, Quality

## Abstract

**Background:**

Osteoporosis is a complex disease with multiple causes and treatments, making access to accurate online health information (OHI) critical for informed decision-making amid rising digitization and misinformation. This study aimed to evaluate whether current OHI on osteoporosis meets the criteria for evidence-based health information (EBHI) to support informed decisions.

**Methods:**

We conducted a descriptive cross-sectional study to evaluate the quality of OHI in German and English. The search terms “osteoporosis” and its German translation were used in Google searches, which were independently screened by two reviewers. An initial exploratory search covering the first five result pages in each language was conducted in March 2021, followed by an extended search in September 2021 that included the first ten pages of results. Websites were included if they targeted laypeople, offered at least two options (including waiting or doing nothing), and were presented in a coherent way. Quality was assessed using the validated “Mapping the Quality of Health Information” (MAPPinfo) checklist. To develop a shared understanding of how the criteria should be applied to the specific health problem of osteoporosis, a subset was independently assessed by two raters, with the remaining assessments conducted individually. Data analysis included the calculation of mean scores (0–100%), which represent the compliance with the EBHI criteria.

**Results:**

146 websites met the inclusion criteria (81 German, 65 English) and came from various sources, including pharmaceutical companies (30%), hospitals (35%), government agencies (16%), specialist organisations (10%), non-profit organisations (8%), patient organisations (4%), health insurances (3%), and other providers (4%). Websites covered OHI on diagnostics (*n* = 120), treatment (*n* = 136), prevention (*n* = 102), and rehabilitation (*n* = 1), with 92% addressing multiple areas. On average, the information quality of the OHI is 15.3% (SD = 5.2), 16.7% (SD = 5.7), and 17.4% (SD = 5.6) for diagnostics, treatment, and prevention, respectively.

**Conclusions:**

Current OHI on osteoporosis often fails to meet basic standards of EBHI, highlighting the urgent need to improve the quality to support informed decision-making. As the use of OHI increases, ensuring that these resources are reliable and evidence-based is critical to improving patient outcomes.

**Clinical trial number:**

Not applicable.

## Introduction

 Osteoporosis is a complex, multifactorial disease with different causes and treatment options [1]. In particular, patients with chronic or complex conditions like osteoporosis may benefit from internet-based health information [2, 3]. Accordingly, the importance of online health information (OHI) about osteoporosis seems to increase. Since 2015, Google hits for the term “osteoporosis” have been rising constantly [4]. When it comes to health-related decisions, the majority of people wants to be actively involved in the decision-making process [5–7]. This can be especially crucial concerning complex diseases with different treatment options. Dissatisfaction with the information provided in consultations can result in patients seeking information independently [8]. Although physicians and family members are recognised as the most important sources of information, a growing number of people are using the Internet for health-related topics [9, 10]. Online search patterns vary depending on individual characteristics, including age, gender, socioeconomic background, and level of education [8]. The extensive availability of OHI offers the opportunity to support informed decision-making [2]. As a potential source of information independent of a medical consultation, OHI can provide laypeople with evidence-based health information (EBHI), which is a prerequisite for informed decision-making [11]. Accordingly, the provision of EBHI is advocated at the national and international level [12–15]. Various aspects, such as the information on the health problem, its natural course and the presentation of risks and benefits, should be included in evidence-based health information to enable an informed choice. There are many different interest-holders that provide health related information on the internet and its quality and content can vary substantially. The chances of misinformation are evident [16]. Inadequate health literacy is a problem that is repeatedly highlighted in the literature. This is because people often have difficulty accessing, interpreting and using health information effectively to make informed decisions. It is not only important to increase health literacy, but also to provide high-quality information [17].

The aim of this study was to investigate whether current OHI on osteoporosis meets the criteria of EBHI and thus fulfils the requirements necessary to enable patients to make an informed decision.

## Methods

We assessed the quality of German and English OHI on osteoporosis using a cross-sectional descriptive design.

### Search Procedure

The aim of the search for websites on the topic of osteoporosis was to identify OHI that patients or other interested people could also find through independent searches. The search was carried out using the Google search engine, chosen because it was by far the most frequently used search engine [18]. For the search the browser settings were reset and the private mode was used. We used “osteoporosis” as a search term in the German and English languages. An initial orientating search for an overview of the information landscape was carried out in March 2021 in preparation for a symposium. The results of the first 5 pages for each language were extracted. In September 2021, the search was updated and expanded to include the results from the first 10 pages for each language. The websites were extracted to an Excel spreadsheet and J.L. and S.Z. independently checked for inclusion and exclusion criteria. We included OHI that (1) addressed laypeople, (2) presented at least two options (including waiting or doing nothing) regarding diagnostic, therapy, prevention or rehabilitation and (3) was presented in a coherent way. The coherence of OHI was defined on the basis of their structure. It is considered that a system of hierarchically organised pages from the same provider constitutes a single OHI. Conversely, a referral to another provider is not considered to be part of the same OHI. Websites that were labelled as advertising by Google, were not publicly accessible and OHI that contained only videos was excluded. Duplicates were removed.

### Data collection

OHI were categorised on the basis of four variables: provider, language, country of origin and subject area.

The providers were divided into eight categories, including commercial providers, hospitals and medical practices, specialist organisations, government and government-funded providers, non-profit organisations, patient organisations, health insurance companies and other providers. As German-speaking and English-speaking OHI can be created in different countries, a further subdivision was made according to the OHI’s country of origin. If a clear classification was not possible, the health information was categorised as “unclassifiable”. With regard to the subject area, a distinction was made as to whether diagnostics, treatment, prevention or rehabilitation were addressed. The allocation of the individual contents to these subject areas was based on the structure or description of the OHI itself.

### Quality assessment

For the quality assessment, we used the “Mapping the Quality of Health Information” (MAPPinfo) checklist [19]. MAPPinfo is a validated checklist that operationalises the quality concept of the “guideline EBHI” [20] which in turn was based on the Good Practice Guidelines for Health Information [21] and the criteria for evidence-based patient information [22]. By providing a comprehensive operationalisation of the criteria and presenting best practice examples, the checklist allows people without specialised training or expertise in evidence-based practice to critically evaluate the quality of health information. It demonstrates good inter-rater reliability (mean T coefficient = 0.79). The checklist comprises 19 items for the assessment of four aspects of quality: (1) Definition: Description of the target group and the aim of the information. (2) Transparency: Production of the information (e.g. authors, financing, topicality, sources). (3) Content: Content of the information (e.g. explanation of options, presentation of benefits and harms). (4) Presentation: Appropriate presentation of the content. Eleven items are presented in a dichotomous response format (met/not met) and eight items are presented in a trichotomous response format (met/partially met/not met). For the initial search, the quality assessment was carried out by two authors (J.L. and S.Z.). In a first step, five pieces of OHI were independently assessed by both raters. Disagreements were discussed in order to develop a common understanding of how to apply the MAPPinfo criteria to the specific health problem of osteoporosis. Further 64 pieces of OHI were independently assessed. For the updated search, the quality assessment was carried out by S.Z. and a research assistant (B.L.). Again, in a first step five pieces of OHI were independently assessed by both raters. The remaining 72 pieces of OHI were then assessed by only one rater. Any uncertainties that arose were checked by S.Z. and, if needed, discussed with J.L. The ratings were recorded in an early version of the MAPPinfo Evaluation Toolkit based on an Excel matrix [23]. The toolkit enabled the documentation of ratings, the generation of summary tables for the assessment and the creation of diagrams for the visualisation of results.

### Data analysis

Analyses were performed using the MAPPinfo Evaluation Toolkit for each OHI subject area (diagnostics, treatment, prevention and rehabilitation). We calculated a mean score for all items and a corresponding total mean score (SD) indicating the criteria compliance. 100% indicates full compliance with the quality criteria of the guideline EBHI. Based on the MAPPinfo manual, four items representing both content and presentation (Items C4/P1 - C7/P4) were included in the calculation with double weighting. Moreover, the total number of relevant items depends on the content of the OHI. Item C7/P4 is only used for diagnostic problems and item P7 only when graphs are used to present frequencies. In addition, mean scores (SD) (range 0 to 100%) were calculated for all four subcategories of MAPPinfo (definition, transparency, content, presentation).

## Results

**Fig. 1:**
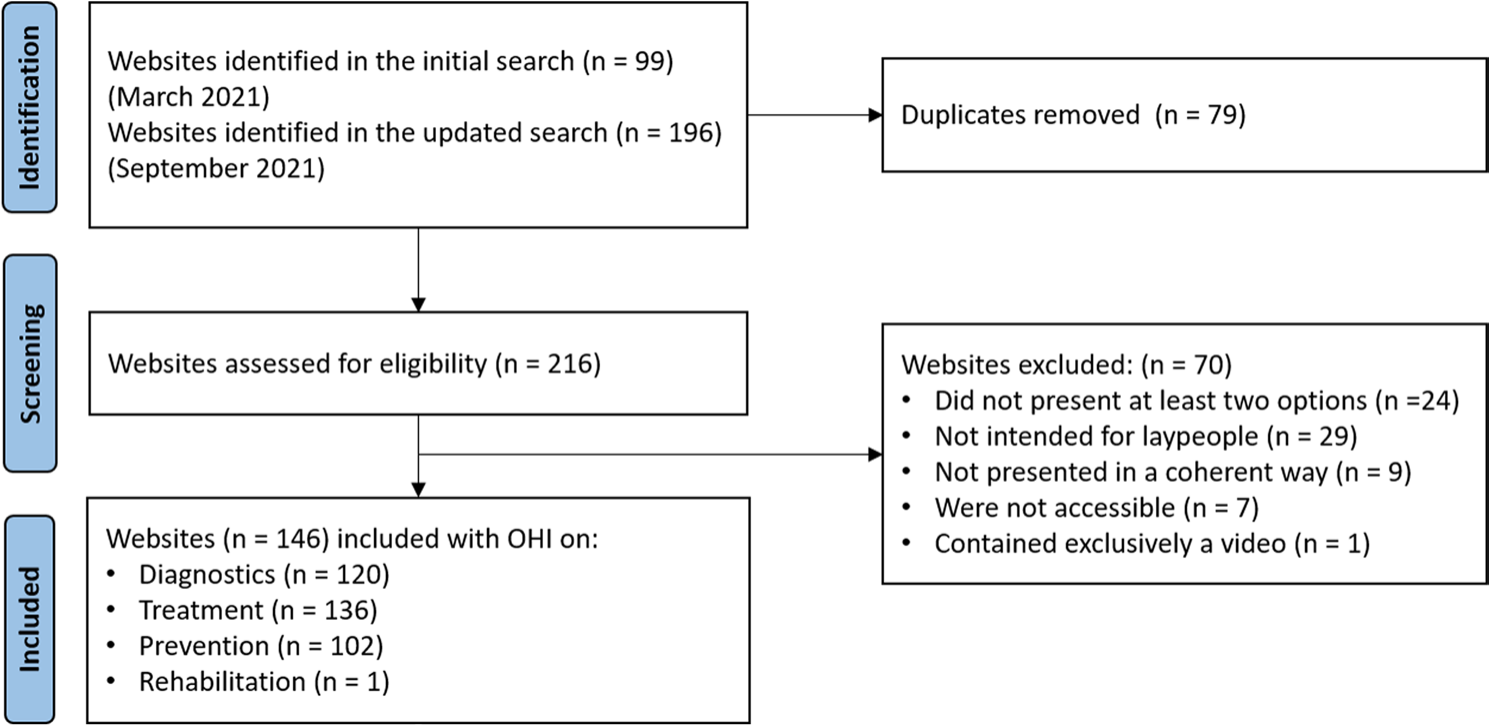
Flow diagram internet search

After removing duplicates, we were able to identify a total of 216 websites. Of these, 117 were in German and 100 in English. After review of the inclusion and exclusion criteria, we were able to include 146 of the websites in the OHI quality assessment. Of these websites, 81 were in German and 65 in English. Please see Fig. 1 for the flow diagram.

OHI was offered by pharmaceutical companies and commercial providers (30%, *n* = 44), hospitals and doctors’ practices (25%, *n* = 37), government and government-funded providers (16%, *n* = 24), specialist organisations (10% *n* = 14), non-profit organisations (8% *n* = 11), patient organisations (4%, *n* = 6), health insurances (3% *n* = 4) and other providers (4%, *n* = 6). 48% (*n* = 70) of the included OHI came from Germany, 28% (*n* = 41) from the USA, 5% (*n* = 8) from the United Kingdom, 4% (*n* = 6) from Switzerland, 3% (*n* = 4) from Australia, 3% (*n* = 5) from Canada, 2% (*n* = 3) from Austria, 1% (*n* = 1) from New Zealand and 5% (*n* = 8) were unclassifiable.

**Fig. 2:**
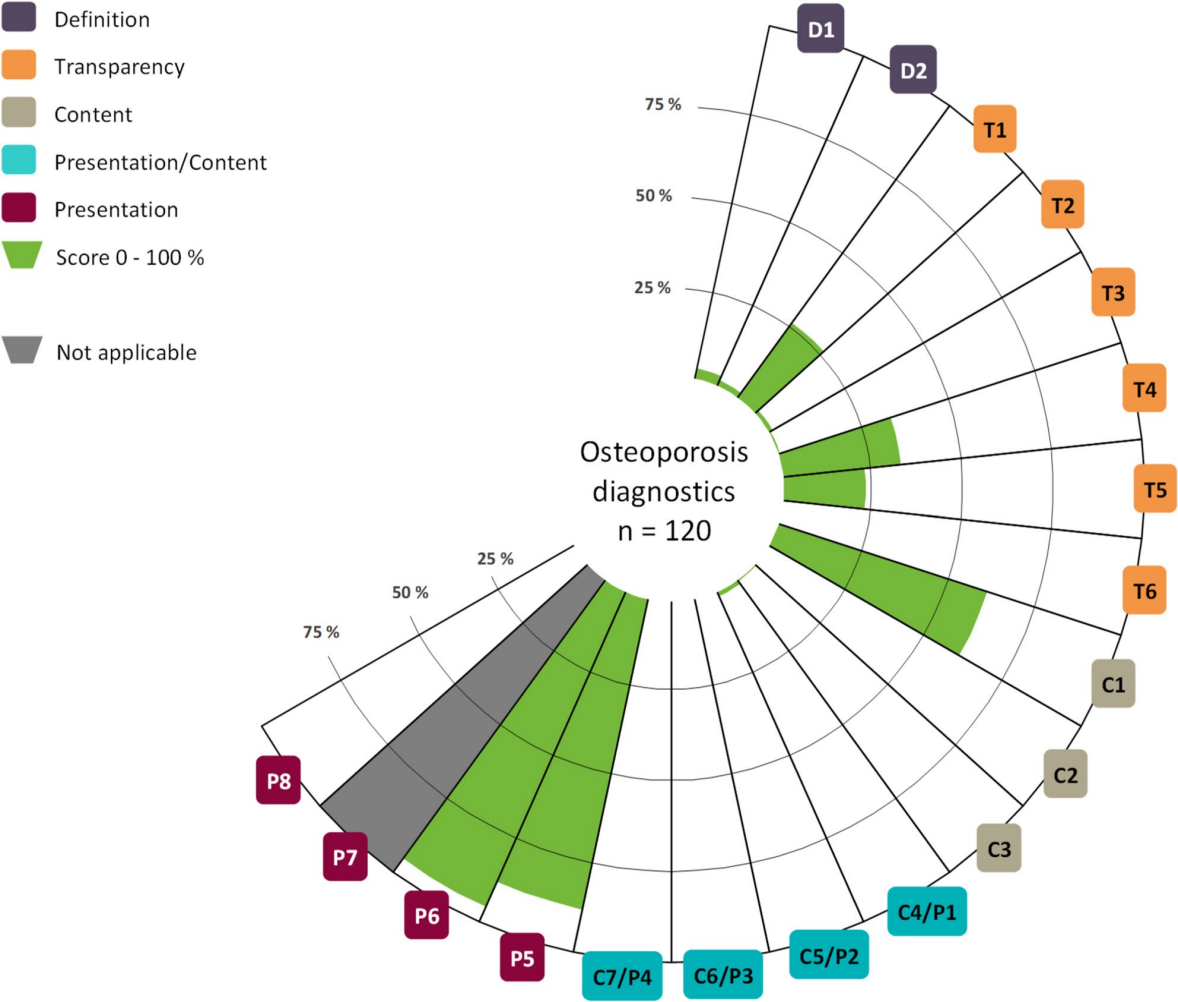
Mean scores per item for the subject area diagnostics

**Fig. 3:**
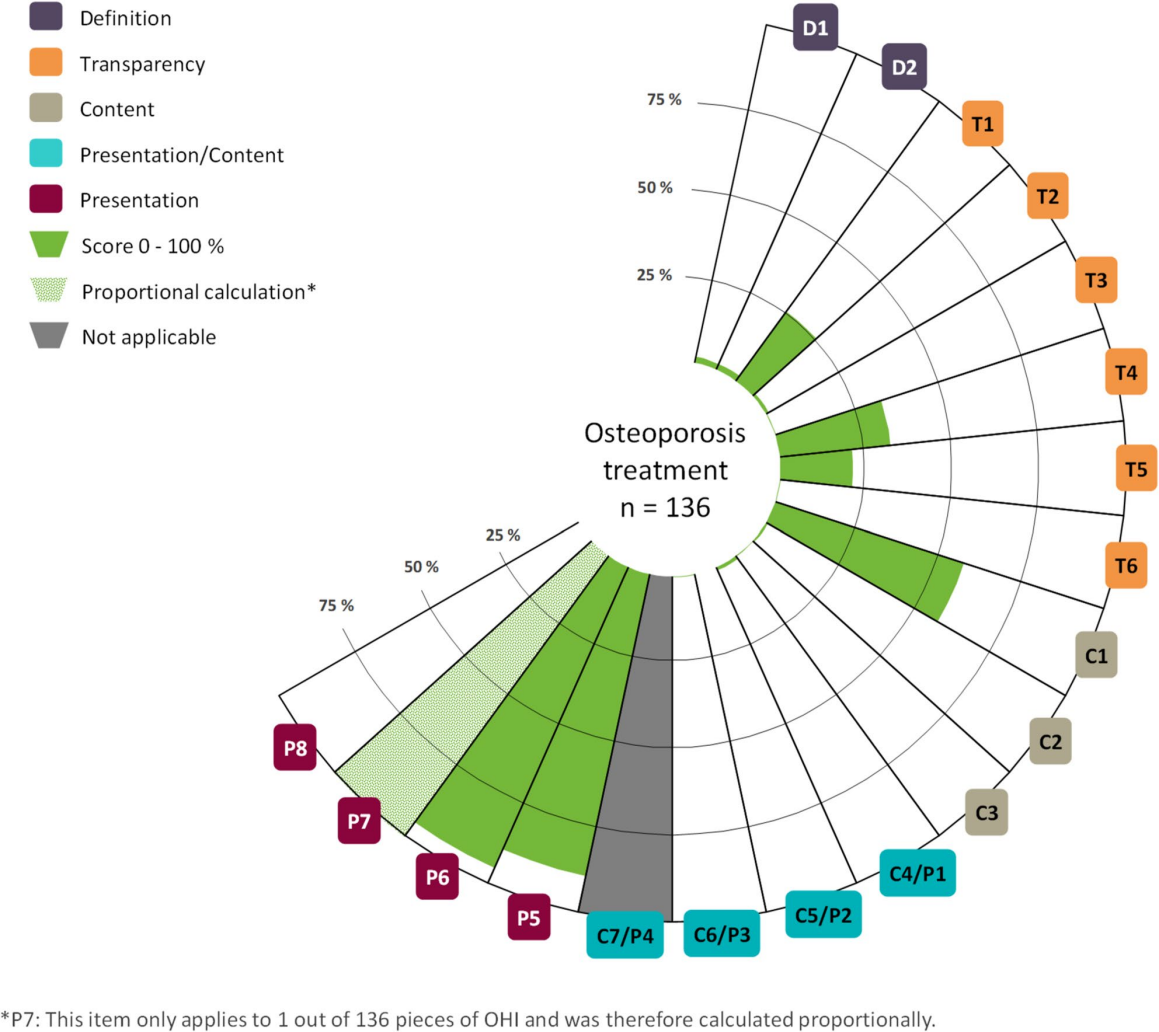
Mean scores per item for the subject area treatment

**Fig. 4:**
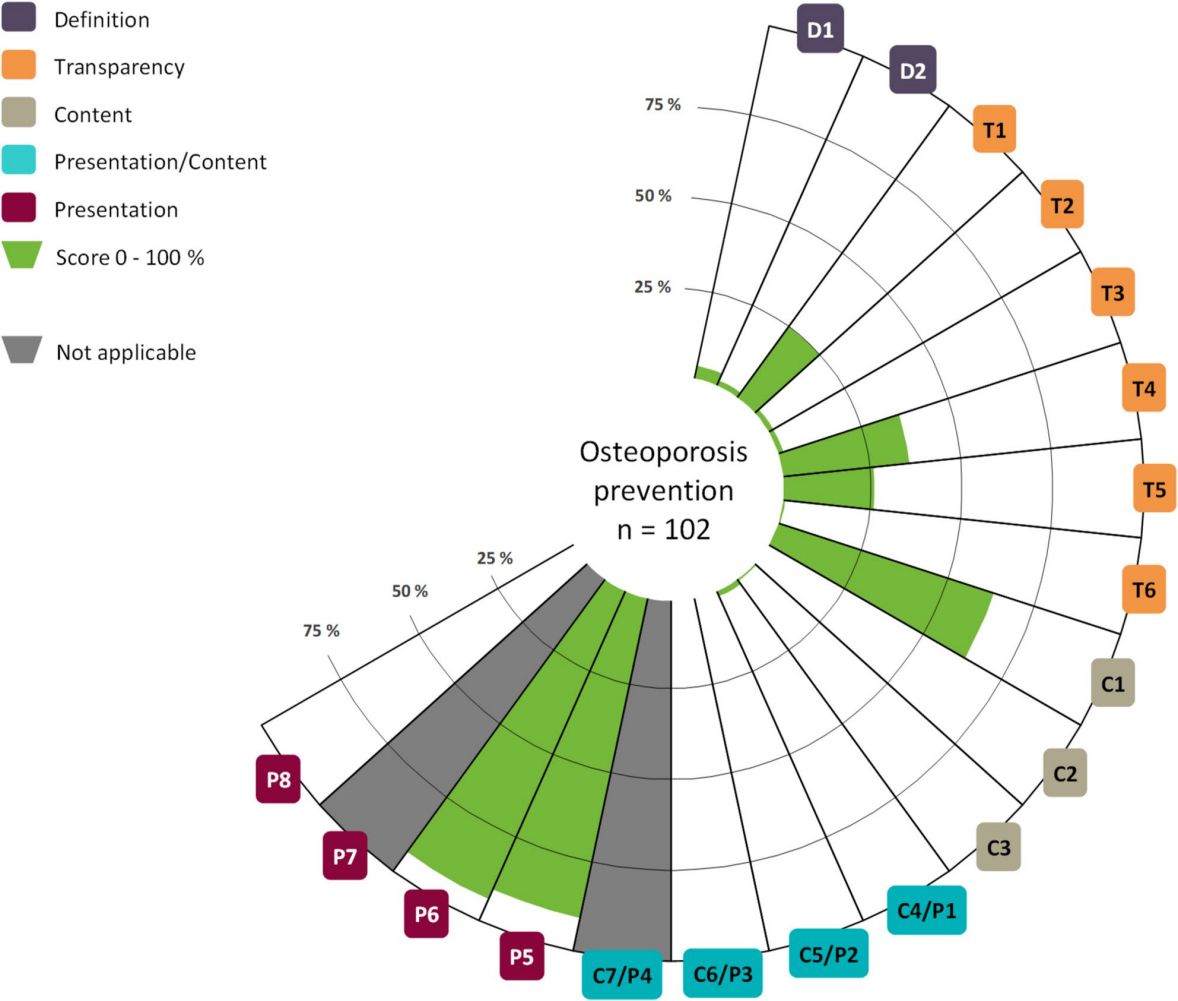
Mean scores per item for the subject area prevention

The websites contained OHI on different subject areas: diagnostics, treatment, prevention and rehabilitation. 92% (*n* = 135) contained OHI on two or more subject areas. 82% (*n* = 120) offered content on diagnostics, 93% (*n* = 136) on treatment and 70% (*n* = 102) on prevention of osteoporosis. Only one website included OHI on rehabilitation. The OHI on rehabilitation received a mean score of 10.0% and fulfilled only 2 items in total. The information quality of the OHI is, on average, 15.3% (SD: 5.2) for diagnostics, 16.7% (SD: 5.7) for treatment and 17.4% (SD: 5.6) for prevention. None of the OHI met all criteria and only three items were rated with a mean of more than 50%. Figures 2, 3 and 4 provide a general overview of the quality of the OHI and the item mean scores per subject area. For detailed information, please see Table 1.

**Table 1:**
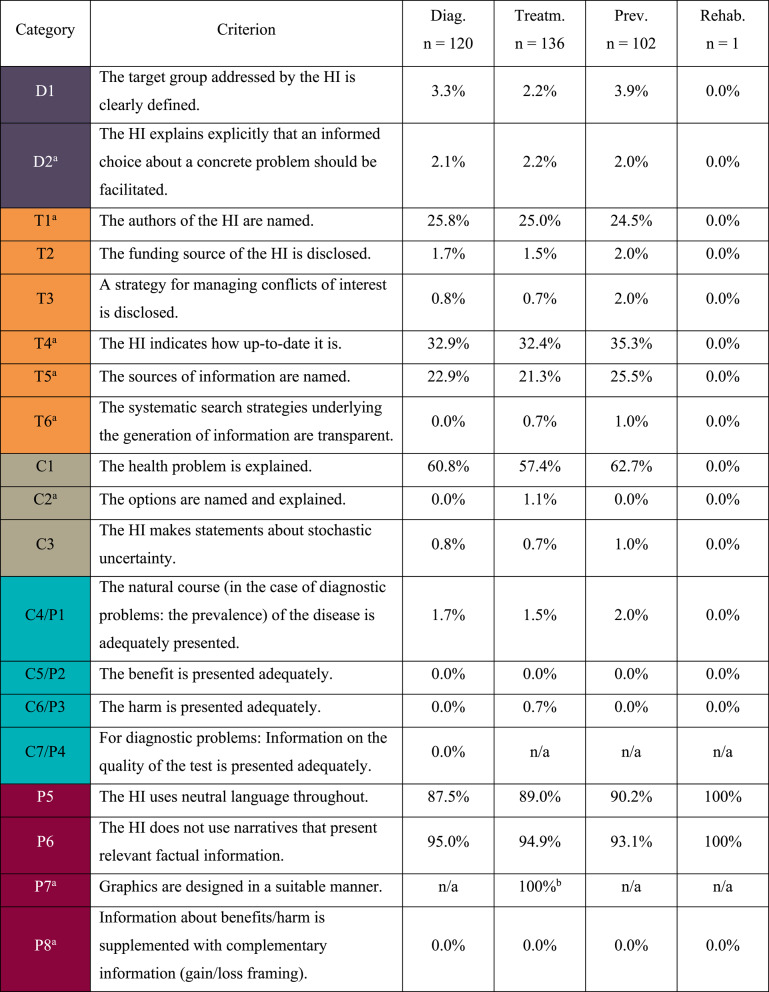
MAPPinfo mean scores (0-100%) per item, per subject area

*HI* Health Information, *D* Definition, *T* Transparency, *C* Content, *P* Presentation, *Diag.* Diagnostic, *Treatm.* Treatment, *Prev.* Prevention, *Rehab.* Rehabilitation, *n/a* Not applicable

^a^ Trichotomous response format (met/partially met/not met), ^b^ Proportionally calculated for 1 out of 136 pieces of OHI

The item scores appear to be mostly comparable across the three subject areas. While the benefit (C5/P2) was not correctly presented by any OHI, one OHI on the subject area of treatment adequately presented possible harm (C6/P3). Item P7 assesses the adequate use of graphics and is only applicable if graphics are used to present benefits or harms in the OHI. In total, only one graphic was used, on the topic of treatment, and was adequately designed, achieving a score of 100%. Item C7/P4 assesses the representation of test quality and is therefore only applicable to the subject area of diagnostic. However, no OHI met the criterion. Regarding the quality categories (definition, transparency, content and presentation), there seem to be no major differences between the subject areas of diagnostic, treatment and prevention. However, we found clearly higher mean scores for presentation than for the other categories. See Table 2 for details.

**Table 2:** Results per quality category and subject area

	Diagnostics	Treatment	Prevention	Rehabilitation
Total				
n	120	136	102	1
Mean (0-100)	15.3%	16.7%	17.4%	10.0%
SD	5.2	5.7	5.6	/
Minimum	4.5%	5.0%	5.0%	/
Maximum	31.8%	38.1%	35.0%	/
Definition				
n	120	136	102	1
Mean (0-100)	2.7%	2.2%	2.9%	0.0%
SD	12.5	11.2	13.3	/
Minimum	0.0%	0.0%	0.0%	/
Maximum	100%	100%	100%	/
Transparency				
n	120	136	102	1
Mean (0-100)	14.0%	13.6%	15.0%	0.0%
SD	11.6	11.4	11.4	/
Minimum	0.0%	0.0%	0.0%	/
Maximum	50.0%	50.0%	50.0%	/
Content				
n	120	136	102	1
Mean (0-100)	9.0%	10.2%	10.9%	0.0%
SD	7.6	9.1	8.9	/
Minimum	0.0%	0.0%	0.0%	/
Maximum	28.6%	41.7%	33.3%	/
Presentation				
n	120	136	102	1
Mean (0-100)	26.3%	31.1%	30.9%	33.3%
SD	6.1	6.9	7.2	/
Minimum	0.0%	0.0%	0.0%	/
Maximum	42.9%	50.0%	50.0%	/

## Discussion

In this study, for the first time, a large amount of OHI on osteoporosis from different countries and in different languages was assessed using a validated tool to determine whether the minimum requirements for making an informed decision were met. Other established tools for assessing the quality of health information do not allow the requirements for an informed decision to be checked [19]. The majority of the OHI assessed covered one or more of the topics of diagnostics, treatment and prevention. The subject area of rehabilitation was only covered by one website. The overall mean scores for compliance with the “guideline EBHI” criteria are low for all subject areas. None of the OHI was able to fulfil all criteria of the MAPPinfo checklist. The results indicate that the OHI currently available on Google does not fulfil the criteria of the “guideline EBHI” and does not support informed decision-making.

MAPPinfo is intended to be used as a checklist to assess compliance with current guideline recommendations for EBHI. The criteria listed in this guideline are considered essential for making an informed decision. There are still several aspects that make up good health information, but these are not included in this checklist, as no clear recommendation can yet be made for them from a scientific perspective [19]. Furthermore, the content of the information could be evaluated on the basis of current evidence. However, without very specific expertise in the field, this would only be possible on a selective basis, e.g. on the basis of current guidelines.

Three items stand out clearly in the subject areas of diagnosis, treatment and prevention. The item describing the health problem (C1) achieved a mean score of over 50% across the subject areas (diagnosis: 60.8%; treatment: 57.4%; prevention: 62.7%). Even though it is one of the three items that corresponds best with the given criteria, the low compliance is alarming, as understanding the illness is the basis for understanding treatment approaches and diagnostic procedures. The lack of information can lead to uncertainty and misconceptions among patients [24]. The other two items that stand out more positively are P5 (use neutral language) and P6 (do not use narratives), with a mean score of over 80% (diagnosis: 87.5%; treatment: 89.0%; prevention: 90.2%) and over 90% (diagnosis: 95.0%; treatment: 94.9%; prevention: 93.1%) respectively across the topic areas, which is encouraging.

It should be noted that a comparison of the results with studies that also assessed the quality of OHI for osteoporosis is only possible to a limited extent since the other studies used different instruments, some of which were not validated, or the criteria were not operationalised, or the criteria used did not reflect current evidence or ethical requirements. This is supported by a systematic review during the preparation and validation of MAPPinfo, which found that no existing instrument adequately reflects the criteria of the “guideline EBHI” [25]. Nevertheless, some items were similar, so it was meaningful to compare some of the results.

It was surprising that more general aspects of transparency (author disclosure (T1), timeliness (T4) and literature sources (T5), which are easier to fulfil compared to other items) achieved only low mean scores. In terms of authorship transparency (T1) (diagnosis: 25.8%; treatment: 25.0%; prevention: 24.5%), the OHI examined in our studies scored slightly better than that of Lopez-Olivo et al. [26] and roughly identical to that of Fuzzell et al. [27], where about one third of the websites disclosed authorship. More specific transparency disclosures such as funding (T2), the management of conflicts of interest (T3) and the disclosure of research strategy (T6) were hardly covered by the OHIs. However, this entails a risk of bias. If these facts are not disclosed, it is impossible for users to judge this question. Regarding the disclosure of the source of funding (T2), our results are in contrast with those of another study in which at least one third of OHI disclosed it [26]. This discrepancy could possibly be due to different operationalisations. To fulfil the MAPPinfo criterion, it is necessary for financial contributions to be directly attributed to an OHI; a general list of sponsors is not sufficient, as the influence of different sponsors on different pieces of OHI may be different.

The items in the definition category (D1, D2) are not reported satisfactorily in terms of the “guideline EBHI” criteria across all subject areas, so users cannot know whether the information provided applies to them at all. With a disease such as osteoporosis, which can have a variety of causes and requires individualised treatment, it is problematic if the target group of the information is not defined. Only one OHI fulfils both definition criteria by formulating the target group of the information and the goal of an informed decision. These findings are consistent with those of Crawford-Manning et al., who found in a smaller sample that none of the analysed OHI specified the target population precisely [28]. Lopez-Olivo et al. reported that a larger proportion (22.9%) of OHI declared the target population [26]. It is important that users themselves can recognise whether this OHI applies to them or when they should be given further information to clarify issues, and users want to know this [29]. This can only be achieved if the target group is clarified separately for each OHI. It is not sufficient for the MAPPinfo criterion to be fulfilled if there is a general reference to the target group for the entire website and all the OHI it contains.

The compliance of the content criteria, with the exception of the mentioned item (C1 explanation of the health problem), was generally low and in some cases not addressed at all or only by individual pieces of OHI. The listing of all possible options, including doing nothing as a choice, is essential information when it comes to individualised consideration of options in accordance with individual preferences and values. Lopez-Olivo et al. reported similar findings in relation to the disclosure of the possible effects of non-treatment [26].

The benefits and harms of the various options are not adequately presented by any OHI, except for one OHI that adequately presents harms for treatment. This is particularly problematic for drug treatment options, as it is known from the literature that many people with osteoporosis have concerns about potential and actual side effects of medication and want to know the benefits [24]. If the potential benefits and harms are not adequately presented, it is impossible for users to make an informed decision and weigh up the options. Benefits and harms are adequately presented if quantitative data for patient-relevant outcomes are presented in absolute frequencies and with a standardised reference value or, alternatively, if explanations of uncertainty are presented. The balanced presentation of benefits and harms, as used as a criterion in other studies [26, 28], is in our view not sufficient without frequency data. In the study by Fuzzell et al. evaluating OHI on bisphosphonates, 1.8% (*n* = 4) reported benefits and 2.6% (*n* = 6) reported side effects with absolute frequencies (e.g. X out of 100) [27], not taking a complete presentation of benefits and side effects into account. This indirectly corresponds to the C5/P2 or C6/P3 criterion of MAPPinfo, which rejects the use of verbal descriptors alone and instead favours the use of absolute frequencies. To fulfil the MAPPinfo criterion, the quantification or uncertainty information must be presented for each reported patient-relevant outcome. It cannot be assumed that an incomplete presentation of benefits and harms is superior to a missing presentation.

In addition, information on benefits and harms can be conveyed by formulations that are either positive (gain framing: e.g. number of survivors after five years) or negative (loss framing: e.g. number of deaths after five years). Both types of wording should be provided. This avoids a possible direct influence on the users. None of the analysed OHI used appropriate gain/loss framing (P8).

Similar analyses of the quality of health information with regard to the fulfilment of the EBHI criteria were conducted in Norway. In 64 cross-sectional studies, 1948 pieces of health information from 16 public health domains were evaluated using the MAPPinfo checklist. The results of this study similarly revealed an average quality of 22%, indicating that Norwegian health information is inadequate for facilitating an informed choice. The overall mean and the mean of individual criteria are comparable to our results, suggesting that deficits in compliance with the “guideline EBHI” are widespread in all areas of health information [20].

### Strengths and limitations

One of the strengths of this study is the use of a validated tool specifically designed to evaluate the quality of health information. This lends credibility to the results and ensures that the assessment is based on a clearly defined standard. In addition, this work contributes to the challenge of reducing misinformation, which is very common in OHI [16]. It provides more transparency regarding the quality of OHI relating to osteoporosis. In this respect, this study addresses a highly relevant and current topic in times of increasing digitalization and growing demand for OHI. We were able to collect a considerable amount of OHI from different English and German-speaking countries, which provides a broader perspective on the quality of publicly available information and expands the applicability and relevance of the results for different populations, compared to other studies that have examined the quality of osteoporosis OHI and focused only on English-language information.

This study also has some limitations. We did not include a systematic patient or public involvement. However, as part of the study, we discussed some of the results with interest-holders at a symposium. This could be an approach in further studies to explore the significance of the lack of information quality for patients and interest-holders on the one hand and to systematically pilot test the comprehensibility of the MAPPinfo visualisation on the other. Furthermore, the online content itself is frequently updated, changes may not have been maintained for some time or may no longer be available at this time. As such, the timeliness of our ratings may no longer reflect the current status. However, mere updates or changes are unlikely to produce significant improvement. Meeting the MAPPinfo criteria will require a fundamental shift in mindset among health-information providers and targeted training in the development of evidence-based patient materials. We are not aware of any programmes in the interim that could have yielded substantially better information quality. In addition, only the Google search engine was used to search for OHI. Other search engines that could also be used to search for information were not taken into account. However, Google’s search engine is by far the most popular [18]. Nevertheless, it is possible that other search engines may have different top ranked OHI. Our search strategy is also limited in the search terms used and the number of results pages screened. No synonyms for osteoporosis were used, and only the first ten pages were screened. Therefore, we might have missed some information. However, this search strategy most closely reflects the approach taken by users, and our study therefore likely includes the information that users would also have found. The MAPPinfo checklist is limited to ethically and scientifically legitimate criteria that are actually supported by evidence. Accordingly, the checklist does not ask about other potentially important aspects of EBHI that have not yet been scientifically investigated. Rather, the criteria presented in this checklist should be considered as a minimum requirement for an informed decision. Potential components of EBHI that can only be assessed with background information are not covered by this checklist. The study included only OHI that presented at least two options, thus giving users the impression that they could make an informed decision based on the information provided. OHI that offered only a single option was excluded. This exclusion may limit the results, as users without a clearly defined objective may not recognize the intended purpose of OHI and could still rely on single-option information to make decisions. The findings of this study indicate that the excluded websites may be less compliant with EBHI standards, which may result in an overestimation of the quality of OHI on osteoporosis. Future quality studies should weigh the pros and cons of this selection criterion carefully.

## Conclusion

Currently, OHI on osteoporosis does not meet essential standards of EBHI and consequently does not present added value to facilitate informed decision-making. Considering the increasing importance and use of OHI, this study could reveal an urgent need for action to improve the quality of osteoporosis-related OHI. The dissemination of the MAPPinfo checklist as a template to develop sound EBHI can be useful within this context, as it can serve for different purposes. First, it can be used as a guide for health information developers, as it lists essential evidence-based aspects of an EBHI. Second, it can be used as a tool for assessing existing health information by untrained health science professionals. The visualisation of the compliance of individual OHI with the MAPPinfo criteria could help users to assess in which areas the OHI provides good information and in which areas this information is not sufficient. Good health information can thus be specifically selected and compared with others so that decisions can be based on reliable information if available. However, more research on what constitutes good health information in terms of informed decision-making is needed and thus also the MAPPinfo checklist needs further development as well.

## Data Availability

The datasets used and/or analysed during the current study are available from the corresponding author on reasonable request.

## Abbreviations

EBHI: Evidence-based health information
MAPPInfo: Mapping the Quality of Health Information
OHI: Online health information
